# Age Group Differences in the Awareness of Lifestyle Factors Impacting Cardiovascular Risk: A Population-Level Study

**DOI:** 10.7759/cureus.41917

**Published:** 2023-07-15

**Authors:** Okelue E Okobi, Ayomide H Adeyemi, Patience N Nwimo, Onyinyechukwu B Nwachukwu, Ure K Eziyi, Cynthia O Okolie, Georgenia Orisakwe, Faith A Olasoju, Omouyi J Omoike, Linda Nkechinyere Ihekire, Jeffrey Afrifa-Yamoah

**Affiliations:** 1 Family Medicine, Medficient Health Systems, Maryland, USA; 2 Family Medicine, Lakeside Medical Center, Belle Glade, USA; 3 Medicine, VN Karazin Kharkiv National University, Kharkov, UKR; 4 Internal Medicine, Richmond Gabriel University, Belair, VCT; 5 Internal Medicine, American International School of Medicine, Georgetown, USA; 6 Public Health, Liverpool John Moores University, Liverpool, GBR; 7 General Physician, University of Lagos, Lagos, NGA; 8 Internal Medicine, University of Nigeria College of Medicine, Enugu State, NGA; 9 General Medicine, Madonna University, Elele, NGA; 10 Family Medicine, People's Friendship University Russia, Moscow, RUS; 11 Medicine and Surgery, University of Ottawa, Ottawa, CAN; 12 Internal Medicine, Windsor University School of Medicine, Cayon, KNA; 13 Internal Medicine, Korle Bu Teaching Hospital, Accra, GHA

**Keywords:** alcohol, awareness, cardiovascular, smoking, lifestyle

## Abstract

Objective: This study evaluated the age-group differences in the levels of awareness of cardiovascular lifestyle risk factors.

Methods: Data from 5,219 individuals were analyzed. Age was classified as young, middle-aged, and older adults. Lifestyle factors included smoking, exercise, noting calorie information, and alcohol. The Chi-square (Χ2) test was used to analyze age-group differences in awareness. Multiple logistic regression was used to examine the relationship between age group and level of awareness.

Results: Awareness of at least one lifestyle factor was highest in middle-aged adults at 47.8% (2232/5095), followed by young adults at 32.1% (991/5095) and older adults at 20.1% (1872/5095). The older age group was linked to an increment in the odds ratios (AOR: 1.47, CI: 1.06 to 2.03) of being aware of exercise recommendations. There was a significant association between the middle-aged (AOR 0.74, CI: 0.56 to 0.99) and older-aged (AOR 0.76, CI: 0.58 to 0.99) categories with reduced odds of individuals becoming increasingly aware of the calorie information found in various menu boards and food menus.

Conclusion: Middle-aged adults were the most aware of lifestyle risk factors. Middle-aged or older adults are associated with being less aware of calorie information on food menus and menu boards. Further research should evaluate the reasons behind low levels of awareness in younger adults.

## Introduction

Cardiovascular diseases are among the leading causes of mortality in the United States (US), with factors including unhealthy diets, a lack of physical activity, cigarette use leading to nicotine addiction, and unhealthy alcohol consumption contributing to their prevalence [1-3]. Regarding the sociodemographic factors associated with these lifestyle factors, studies have shown that individuals in racial minority groups and those in low socioeconomic classes are less likely to adhere to healthy eating habits, meet exercise recommendations, and are more likely to consume unhealthy amounts of alcohol and be addicted to nicotine from cigarettes [4,5]. To address these disparities, multiple programs have increased awareness of modifiable lifestyle factors over the years [6-8].

Studies analyzing the effectiveness of various awareness programs have been conducted in isolated groups. For instance, among nurses, pregnant women, and individuals at risk of cancer, 45%-70% were aware of the negative effects of alcohol [9-13]. A similar level of awareness of the effects of smoking cigarettes was found in a group of current smokers aged 18 to 54 [13]. Regarding awareness of exercise recommendations, different populations have shown low levels of awareness. In a UK study, about 18% of the population was aware of appropriate exercise recommendations [14]. Similar results were seen in a small study in the US [15]. In terms of increased awareness of caloric information found on food items, there have been varied and confusing reports observed across diverse age groups. A recent systematic literature review has revealed low perceptions of food labeling information by both older and younger adults, although research conducted by Jackey et al. disclosed increased utilization and perception among older adults [16].

Due to the variations in the levels of awareness of these lifestyle factors across diverse populations and the relatively high prevalence of cardiovascular diseases in middle- and older-aged adults, it is important to assess age-group awareness of these lifestyle factors. The objective of the present study is to evaluate age-related differences in the levels of awareness of lifestyle factors that increase cardiovascular risk and the existing association between the age group to which an individual belongs and the awareness of these lifestyle factors.

## Materials and methods

Study design and population

The study mainly involved a review of cross-sectional data acquired from the Health Information National Trends Survey (HINTS) 5 cycle 3. HINTS 5 cycle 3 refers to the probability-based data drawn from the National Cancer Institute and collected between January 22 and April 30, 2019. It is a nationally representative survey of the US noninstitutionalized adult population. Full details about the HINTS methodology and data collection are available on the HINTS website [17]. The data consisted of 5,219 entries, representing 243,552,645 people; 219 entries were excluded due to missing information regarding overall awareness.

Sociodemographic variables

The sociodemographic variables used in this analysis included gender and sex, education level, age, ethnicity, race, BMI, and income. Age was recorded in the form of age groups, including young adults aged between 18 and 39 years, middle-aged adults with ages ranging between 40 and 64 years, and older adults comprising individuals aged 65 years and above. Sex was coded as male and female, while race was coded as Hispanics, non-Hispanic whites, non-Hispanic blacks, and others. Education level was coded as above college education, college graduate, some college education, and no college education. Income was further coded as below $20,000, between $20,000 and $34,999, between $35,000 and $49,999, between $50,000 and $74,999, and above $75,000. BMI was coded as not obese if it was below 30 Kg/m2 and obese if it was above 30 Kg/m2.

Awareness of lifestyle factors

Awareness of the Effects of Alcohol

"In the last year, how much information have you been told regarding the adverse health outcomes associated with drinking alcohol from either a physician or healthcare professional?" Coded as "yes/no." Yes, if they had heard a little, some, or a lot, and no, if they had neither heard anything nor seen a physician or any healthcare professional in the last year.

Awareness of the Effects of Smoking

"Tobacco nicotine remains the major substance that makes individuals desire to smoke." Coded as "yes/no." Yes, if they strongly agree or agree, and no, if they disagree, strongly disagree, or do not know. "Nicotine addiction is a matter of great concern to me." Coded as "yes/no." Yes, if they strongly agree or agree, and no, if they disagree, strongly disagree, or do not know.

Smoking awareness was coded as "yes/no." Yes, if they understood that nicotine was the major substance found in tobacco that makes individuals have the desire to smoke or if they had concerns regarding nicotine addiction. No, if they had no knowledge that nicotine was the major substance in tobacco that makes individuals develop the desire to smoke or if they were not concerned about nicotine addiction.

Awareness of Exercise Recommendations

We coded "Government recommendations for exercise and physical activity" as "yes or no." Yes, if they had heard about government recommendations regarding physical exercises and were made to either increase or decrease their physical activity, change their type of physical activity, look for more information about the recommendations, or if multiple responses were selected, or if they did not make any changes at all afterward. No, if they had not heard of any government recommendations for physical activities.

Awareness of Information on Calories Found on Food Menu Boards

The researchers coded, "When was the last time you ordered food in a restaurant, either a sit-down or fast food? Did you consider the information on calories placed next to the food you ordered on the menu board or menu?" Yes if they answered yes, and no if they answered no.

Overall awareness of lifestyle factors was coded as "yes" if they were aware of at least one lifestyle factor and "no" if they were not aware of any lifestyle factor.

Statistical analyses

The distribution of age in each age group was determined using the skewness and kurtosis normality tests and histogram plots. The median and interquartile ranges of the respondents in each age group were reported. Chi-square analysis was used to assess survey-weighted sociodemographic characteristic proportions for each age group and survey-weighted population proportions of awareness levels of cardiovascular risk reduction behaviors by age group. The researchers used multiple logistic regression to analyze factors related to levels of awareness. The model included race, level of education, income, and marital status, as these factors significantly varied across the age groups. HINTS 5 cycle 3 uses an intricate sampling design to acquire nationally representative parameter estimates, and therefore, the weights, based on the jackknife replication technique, were applied to obtain accurate variance estimates. A p < 0.05 level was set as the level of statistical significance. The data analysis was performed using Stata 14.0.

## Results

Table 1 below presents the various sociodemographic characteristics of the study sample and population. It includes 5,219 individuals, representing 244 million US adults in 2019. Overall, 20.2% (n = 1,961) were older adults, representing 50 million of the US adult population; 31.5% (n = 1,015) were young adults, representing 78 million; and 43.4% (n = 2,308) were middle-aged adults, who constituted the majority, representing 120 million of the US adult population. Additionally, 50.9% of the overall population were women, and 63.7% were non-Hispanic whites. The median (IQ) age of the groups was 32 (9) for young adults, 55 (7) for middle-aged adults, and 72 (10) for older adults.

**Table 1:** Sociodemographic characteristics

Variable	Outcomes						
		18 to 39 y		40 to 64 y		65+ y	
	Total weighted %	N	Weighted % (95% CI)	N	Weighted % (95% CI)	N	Weighted % (95% CI)
Sex							
Female	50.9	581	49.4	1242	50.8	950	53.5
Male	49.1	365	50.6	905	49.2	803	46.5
Race							
Non-Hispanic white	63.7	544	57.6	1276	62.3	1188	78.3
Non-Hispanic black	11.2	106	9.8	356	13.4	193	7.7
Hispanic	16.6	194	20.4	330	16.5	187	9.9
Others	8.5	115	12.2	178	7.8	89	4.0
Marital status							
Never married	35.2	483	68.1	448	23.6	189	12.3
Married	51.1	444	29.9	1234	62.5	884	56.2
Formerly married	13.8	53	2.0	591	13.9	848	31.5
Level of education							
No college	39.6	168	26.8	717	42.9	741	51.7
Some College	30.7	213	37.0	512	28.5	445	26.2
College graduate	17.4	377	23.0	610	16.4	394	11.2
Postgraduate	12.3	225	13.3	437	12.3	340	10.9
Income							
< $20,000	18.2	141	20.3	357	16.0	369	20.2
$20,000 – 34,999	11.0	79	7.7	222	10.0	303	19.4
$35,000 – 49,999	13.5	121	15.2	234	11.5	262	16.1
$50,000 – 74,999	17.5	186	17.7	364	17.3	286	17.8
> $75,000	39.8	422	39.1	937	45.2	431	26.5
Body mass index							
Not obese	66.9	720	68.8	1447	64.3	1335	70.3
Obese	33.1	282	31.2	840	35.8	595	29.7

Most of the participants reported being aware of the effects of nicotine from cigarettes (88.5% [95% CI, 87.0-89.9]), followed by awareness of any government recommendation about exercise (60.9% [95% CI, 58.1-63.5]), awareness of alcohol recommendations (40.1% [95% CI, 37.8-42.4]), and awareness of calorie information on menus (44.4% [95% CI, 42.3-46.6]).

Overall, 97.1% (5,095/5,219) were aware of at least one lifestyle factor. Middle-aged adults (2,232/5,095) had the highest awareness of at least one lifestyle factor across all groups, while young adults (991/5,095) came in second with 32.1%, and older adults (1,872/5,095) came in third with 20.1%. Table 2 below shows the distribution of participants by lifestyle factor awareness. Additionally, middle-aged adults had 49.1% awareness of the effect of alcohol on health, 47.8% awareness of the effects of nicotine from smoking cigarettes, 47.4% awareness of the government's exercise recommendations, and 46.5% awareness of paying attention to calorie information on food menus and menu boards.

**Table 2:** Awareness of cardiovascular risk behaviors by age group

Variable	Outcome						
		18-39 y		40 - 64 y %			65+ y %
		n	Weighted %	n	Weighted %	n	Weighted %
“In the past 12 months, how much have you heard about the negative health consequences of drinking alcohol from a doctor or other health care professional?”							
No		594	31.3	1296	47.6	1196	21.1
Yes		400	32.5	955	49.1	699	18.4
“Are you aware of the dangers of Smoking”							
No		90	21.2	254	56.6	248	22.2
Yes		898	32.7	2005	47.8	1637	16.4
“Government recommendations for exercise/physical activity”							
No		387	33.3	814	49.6	625	17.1
Yes		594	30.7	1426	47.4	1255	21.9
“Think about the last time you ordered food in a fast food or sit down restaurant; did you notice calorie information listed next to the food on the menu or menu board?”							
No		456	27.9	1197	50.0	1138	22.1
Yes		543	35.9	1076	46.5	781	17.6

**Figure 1:**
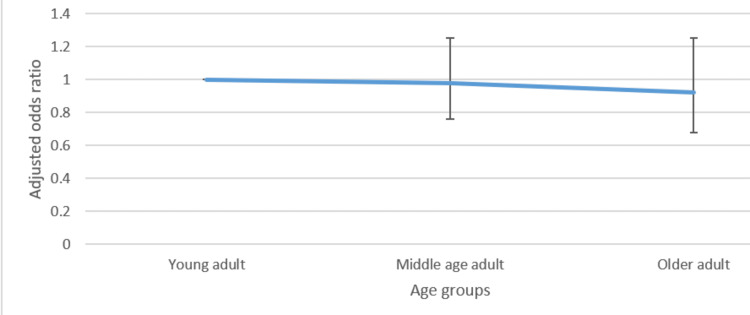
Adjusted odds ratio with confidence intervals of the relationship between age group and awareness of the negative effects of alcohol

**Figure 2:**
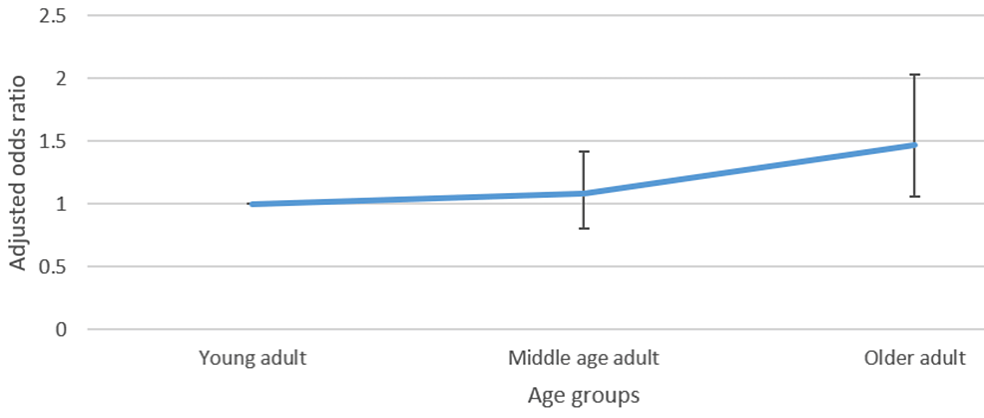
Adjusted odds ratio with confidence intervals of the relationship between age group and awareness of exercise recommendations

**Figure 3:**
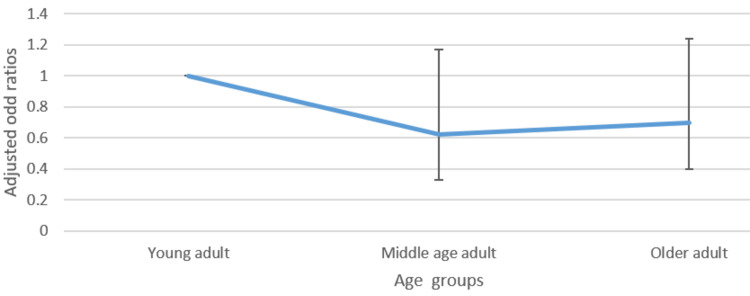
Adjusted odds ratio with confidence intervals of the relationship between age group and awareness of the negative effects of smoking

**Figure 4:**
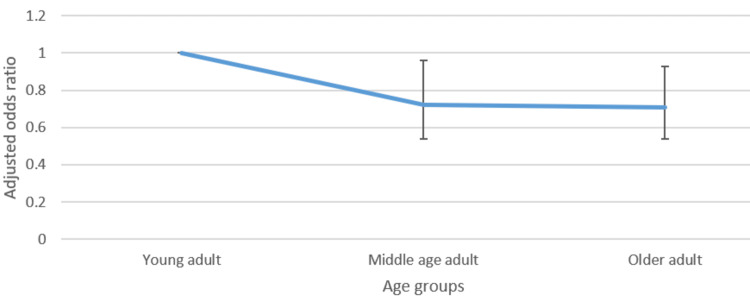
Adjusted odds ratio with confidence intervals of the relationship between age group and awareness of calorie information

Figures 1-4 presented below portray the correlation between varied age groups and the awareness level of such lifestyle factors.

Regarding awareness of the government's recommendation on exercise, belonging to the older age group, compared to the younger age group, was associated with higher odds (1.47, CI: 1.06-2.03) of awareness. However, for calorie information on food items, being a middle-aged or older adult, compared to being younger, was associated with reduced odds (0.72 CI: 0.54-0.96 and 0.71 CI: 0.54-0.93) of being aware of this information. No significant statistical difference between the different age groups was observed with respect to the other lifestyle factors.

## Discussion

The present study aimed to evaluate age group differences in the levels of awareness of lifestyle factors that increase cardiovascular risk and assess the relationship between an individual's age group and awareness of these lifestyle factors. Overall, middle-aged adults were more aware (above 45%) of at least one lifestyle factor compared to young adults (around 30%) and older adults (around 20%). Among the four lifestyle factors, middle-aged adults were most aware of the negative effects of alcohol use, while older adults were least aware. Compared to younger adults, middle-aged and older adults had approximately a 70% probability of being aware of calorie information on food menus and boards.

In this study, although the overall awareness in the population was high, the analysis by age group showed low levels of awareness within each group. Awareness levels decreased from middle-aged to younger and then older adults. Consistent with this result, a study reported relatively higher levels of awareness among middle-aged adults, with factors such as smoking status, weight, and hypertension increasing the odds of awareness [18]. Another study by Lynch et al., which evaluated awareness of lifestyle factors in young adults, found that 30% of these young adults were aware of at least one lifestyle factor, similar to the findings in this study, with black race and low socioeconomic status predicting this low level of awareness. However, it should be noted that Lynch et al. considered other lifestyle factors, such as hypertension and dyslipidemia [19], which may have increased the percentage of those aware due to the higher probability of being aware of at least one of many factors. Additionally, younger adults with borderline cardiovascular risks are less aware [20]. These findings suggest that some existing campaigns aimed at increasing awareness of the risks of unhealthy behaviors have been ineffective among both younger and older adults.

In this study, awareness of the negative effects of various lifestyle factors declined from middle age to older and then younger adults. This is consistent with findings from a population-based study that found middle-aged adults to be more aware of multiple lifestyle risk factors for developing heart disease and cancer compared to younger and older adults [21]. However, Lundborg & Lindgren reported a decline in the perception of the negative effects of alcohol with age [22], which is inconsistent with our results. The reasons why middle-aged adults were mostly aware of the negative effects of alcohol are unclear. In contrast, another study reported that older adults are more likely to see alcohol as less harmful, with a positive effect on the heart [23], which may explain their lower awareness of the negative effects of alcohol.

Regarding calorie information on food menus and menu boards, middle-aged and older adults were less aware of these labels compared to younger adults. Studies in the US and the United Kingdom have shown similar results, with younger adults, female sex, and higher socioeconomic status being associated with higher levels of awareness [24,25]. In contrast to our findings, one study reported a high level of awareness among older adults compared to young adults. However, in that study, poor numeracy was observed among older adults [26]. This suggests that although older adults noticed the information on calories, they might still end up purchasing unhealthy foods. Additionally, younger adults have been shown to be more influenced by calorie content in their decision to purchase an item [27]. Given this positive correlation, adding pictorial information to calorie information on food labels may increase the likelihood of older adults who notice food labels choosing healthier food items.

This study has several strengths. First, HINTS is unique among national databases in its focus on lifestyle factors related to cardiovascular health, providing a large and representative sample size. Second, the stratification of the sample based on age groups is important as cardiovascular disease risk tends to vary by age, allowing for a better understanding of awareness levels of these lifestyle factors.

Despite these strengths, this study also has limitations. First, it is a cross-sectional study design, which means causality cannot be inferred from these relationships. However, the information presented here can inform more rigorous studies estimating risk, and the results can be used in developing evidence-based health promotion programs. Second, questions regarding awareness of smoking effects were not specific to cardiovascular disease but indirectly assessed awareness of cardiovascular risks associated with nicotine use. Caution must be exercised when drawing conclusions from these results. Last, awareness of other risk factors for cardiovascular disease, such as serum cholesterol levels, was not assessed in the HINTS database and could not be included in this study.

## Conclusions

In conclusion, a significant percentage of US adults are aware of at least one lifestyle risk factor that increases cardiovascular risk. Among different age groups, middle-aged adults have the highest awareness levels, particularly regarding the negative effects of alcohol, compared to other age groups. This may be due to the positive perception of alcohol's impact on the heart. However, both middle-aged and older adults are less likely to pay attention to calorie information on food menus and menu boards compared to young adults. Further research is needed to investigate the reasons for the lower awareness of these lifestyle factors among younger and older individuals.
